# Providing holistic care to children with cerebral palsy treated with transnasal neural stem cell transplantation

**DOI:** 10.3389/fped.2023.1297563

**Published:** 2024-01-05

**Authors:** Xiaoyan Li, Mengyao Li, Xixian Qin, Ying Li, Yachen Wang, Chao Han, Shiwei Ni, Xuna Sun, Peipei Dong, Jing Liu

**Affiliations:** ^1^ Stem Cell Clinical Research Center, The First Affiliated Hospital of Dalian Medical University, Dalian, Liaoning Province, China; ^2^ Dalian Innovation Institute of Stem Cell and Precision Medicine, Dalian, Liaoning Province, China

**Keywords:** neural stem cells, cerebral palsy, holistic care, intranasal administration, pediatric nursing

## Abstract

**Objective:**

Holistic care is a key element in nursing care. Aiming at the heterogeneous disease of cerebral palsy, researchers focused on children with cerebral palsy who received transnasal transplantation of neural stem cells as a specific group. Based on establishing a multidisciplinary team, comprehensive care is carried out for this type of patient during the perioperative period to improve the effectiveness and safety of clinical research and increase the comfort of children.

**Methods:**

Between January 2018 and June 2023, 22 children with cerebral palsy underwent three transnasal transplants of neural stem cells.

**Results:**

No adverse reactions related to immune rejection were observed in the 22 children during hospitalization and follow-up. All children tolerated the treatment well, and the treatment was superior. One child developed nausea and vomiting after sedation; three had a small amount of bleeding of nasal mucosa after transplantation. Two children had a low fever (≤38.5°C), and one had a change in the type and frequency of complex partial seizures. Moreover, 3 children experienced patch shedding within 4 h of patch implantation into the nasal cavity.

**Conclusion:**

The project team adopted nasal stem cell transplantation technology. Based on the characteristics of transnasal transplantation of neural stem cells in the treatment of neurological diseases in children, a comprehensive and novel holistic care plan is proposed. It is of great significance to guide caregivers of children to complete proper care, further improve the safety and effectiveness of treatment, and reduce the occurrence of complications.

## 1 Introduction

Cerebral palsy (CP) is a nonprogressive, permanent motor and postural disorder attributed to disorders in the brain development of fetuses and infants (1). Globally, CP is diagnosed in approximately three of every 1,000 live births (2). Children with CP often need to receive traditional treatment and functional rehabilitation care in the hospital; however, it has little effect on improving the children's motor functions and quality of life (3). Stem cell therapy provides a scientific and promising treatment method for patients with CP, which can significantly improve the gross motor and comprehensive functions of children with CP (4, 5).

The remarkable migratory ability of neural stem cells (NSCs) to integrate into damaged areas of the brain provides strong preclinical evidence for the treatment of children with perinatal brain injury (6). It brings hope for neuroregeneration in patients with CP (7, 8). In recent years, many scholars have proven the safety and early efficacy of NSCs clinical trials and have focused on further clinical translation (9, 10). NSC transplantation has the advantages of being non-invasive, easy to obtain, and avoiding first-pass metabolism in the liver. It has now become an effective alternative to lumbar puncture and intravenous injection in the treatment of central nervous system diseases (11–13). To the best of our knowledge, this is the first study to evaluate the safety and effectiveness of transnasal transplantation of NSCs for the treatment of CP (14). The results show that transnasal transplantation is well tolerated and superior in children, reducing the incidence and severity of adverse events. The degree is much lower than that of patients with CP through lumbar puncture, stereotaxic brain, etc (15).

The treatment and rehabilitative care of children with CP requires the involvement of multidisciplinary healthcare providers, but this involvement has been fragmented (16). Highly decentralized care can easily lead to duplication of services and increased medical costs (17). Holistic care, a key element in nursing, emphasizes treating the patient as a whole person (18). Besides paying more attention to the patients, medical staff also need to pay attention to the patient's environment, psychological state, physical factors, and other factors that affect disease recovery. CP is a heterogeneous disease, and such children deserve personalized and holistic care (19). Considering children with CP who are eligible for stem cell transplantation as a specific group, these patients need overall comprehensive care during the perioperative period.

At present, the nursing experience summary about stem cell transplantation is common in patients with bone marrow dysfunction or failure who receive hematopoietic stem cell transplantation (20). Nursing options for transnasal stem cell transplantation to treat neurological diseases have not been reported. This study is based on the team's first international project of transnasal NSC transplantation to treat children with CP (14). A comprehensive and refined care plan was developed based on establishing a multidisciplinary team, the characteristics of transnasal stem cell transplantation, and the needs of children with CP receiving stem cell therapy, thereby improving the effectiveness and safety of clinical research and increasing children's comfort. This study summarizes the nursing process and precautions for 22 children who received transnasal NSC transplantation for the treatment of CP and proposes a comprehensive and novel overall nursing plan for the treatment of children with neurological diseases by transnasal NSC transplantation.

## 2 Materials and methods

Our team conducted a phase I/II randomized controlled clinical trial to evaluate the safety and efficacy of intranasal transplantation of NSCs in the treatment of infantile CP. The study was registered at ClinicalTrials.gov (NCT03005249) and the Medical Research Registration Information System (CMR-20161129-1003) and approved by the Ethics Committee of the Stem Cell Clinical Research Center of the First Affiliated Hospital of Dalian Medical University (LCKY2016-60). The parents or guardians of all the participants signed an informed consent form before participating in the study.

### 2.1 Patients

All patients were strictly screened before entering the group, including disease diagnosis, clinical classification, severity of illness, age, basic diseases, treatment, and rehabilitation, which must meet the admission criteria before participating in the study (Table 1). Between January 2018 and June 2023, 22 children underwent NSC transplantation. Among the 22 children, there were 16 males and 6 females. The average age and body weight of the selected children were 6.30 ± 2.12 years and 19.52 ± 4.51 kg, respectively. Sixteen families lived in northern China, five in southern China, and one in Singapore. The clinical manifestation in the 22 children was moderate to severe paralysis, characterized by spastic CP induced by ischemia and hypoxia. Among them, 14 cases (63.6%) were spastic (quadriplegic). The others were mixed with seven cases (31.8%) of spastic + dyskinetic and one case (4.5%) of spastic + ataxic. Each child underwent intranasal NSC transplantation three times by the same surgical team at an interval of one month (Table 2).

**Table 1:** Inclusion and exclusion criteria.

Inclusion criteria	Exclusion criteria
Neonates with hypoxic-ischemic CP (mainly including neonates with asphyxia and premature infants);	Systemic diseases that possibly influence treatment or patient compliance;
Clinical manifestations of spastic moderate to severe paralysis;	Potentially life-threatening diseases involving various organ systems;
Moderate to severe CP, GMFCS level II–V;	Brain deformity;
Age 3–18 years, either sex;	Abnormal behaviors or mood disorders;
Regular rehabilitation training for more than 1 year;	Allergies to blood products;
No apparent improvements in clinical symptoms more than 3 months;	Current infectious disease;
Provision of signed informed consent by one of the subject's parents or legal guardians before the commencement of this study.	History of craniocerebral operations before screening;
	Penicillin hypersensitivity
	Positive serological tests such as AIDS, hepatitis B virus, hepatitis C virus and syphilis (antigen or antibody);
	Contraindications to MRI;
	Unwillingness to return for follow-up visits;
	Any patient who the investigators feel is unlikely to benefit from the study intervention (eliminated at the screening stage);
	Allergy to anesthetic agents;
	Coagulation disorders;
	Tumors

**Table 2:** Demographical and clinical characteristics of the patients in this study.

Items		Total (*n* = 22)	Percentage/Mean ± SD
Number of patients		22	
Body weight (kg)		19.52 ± 4.51	
Gravidity		37.32 ± 4.3	
Age (years)		6.30 ± 2.12	
Sex	Male	16	72.7
	Female	6	27.3
CP type	SP(q)	14	63.6
	MT(s + d)	7	31.8
	MT(s + a)	1	4.5
GMFCS	II	2	9.1
	III	8	36.4
	IV	7	31.8
	V	5	22.7
MACS	II	10	45.5
	III	3	13.6
	IV	7	31.8
	V	2	9.1

SD, standard deviation; kg, kilograms; SP(q), spastic (quadriplegic); MT (s + d), mixed type (spastic + dyskinetic); MT (s + a), mixed type (spastic + ataxic); GMFCS, gross motor function classification system; MACS, manual ability classification system.

### 2.2 Technology

#### 2.2.1 Setting up a multidisciplinary medical-rehabilitation-nursing team

The multidisciplinary team comprised experts in regenerative medicine, neurology, otorhinolaryngology, rehabilitation, and nursing to formulate perioperative evaluation strategies according to the patient's specific conditions. The members of the neurology department and nursing team jointly evaluated the vital signs, psychological status, and degree of cooperation of the children before the procedure, evaluated the erosion of the nasal mucosa 3 days and 24 h before surgery, and asked the otorhinolaryngologist to guide them if necessary. Relevant preoperative examinations were conducted, including routine hematuria, liver and kidney function, and other blood tests. We focused on the assessment of central nervous system involvement and completed the evaluation of the nervous system scale, electrocardiography (ECG), ambulatory electroencephalography (AEEG), electromyography (EMG), and magnetic resonance imaging (MRI) one week before treatment. Unlike the first transplantation, the second and third transplantations did not need to be rechecked using ECG and MRI. In addition, the scheme for each transplant was the same, and there were no differences. Nurses paid attention to the main complaints and needs of children and caregivers and prepared rescue personnel and articles. The regenerative medicine technician was responsible for completing the preparation of NSCs according to the child's body weight and transporting the stem cell patch to the treatment room on the day of treatment. The otorhinolaryngologist was responsible for completing the nasal transplantation of the stem cell patch, evaluating the children's reactions during the transplantation process, and providing symptomatic treatment. After completing the three transplants, the children were guided by rehabilitation and nursing experts to complete a unified standard rehabilitation training of not less than 6 months, and a follow-up evaluation was carried out at 1, 3, 6, and 12 months after treatment.

#### 2.2.2 Stem cell patch technique for nasal cavity transplantation

**Figure 1:**
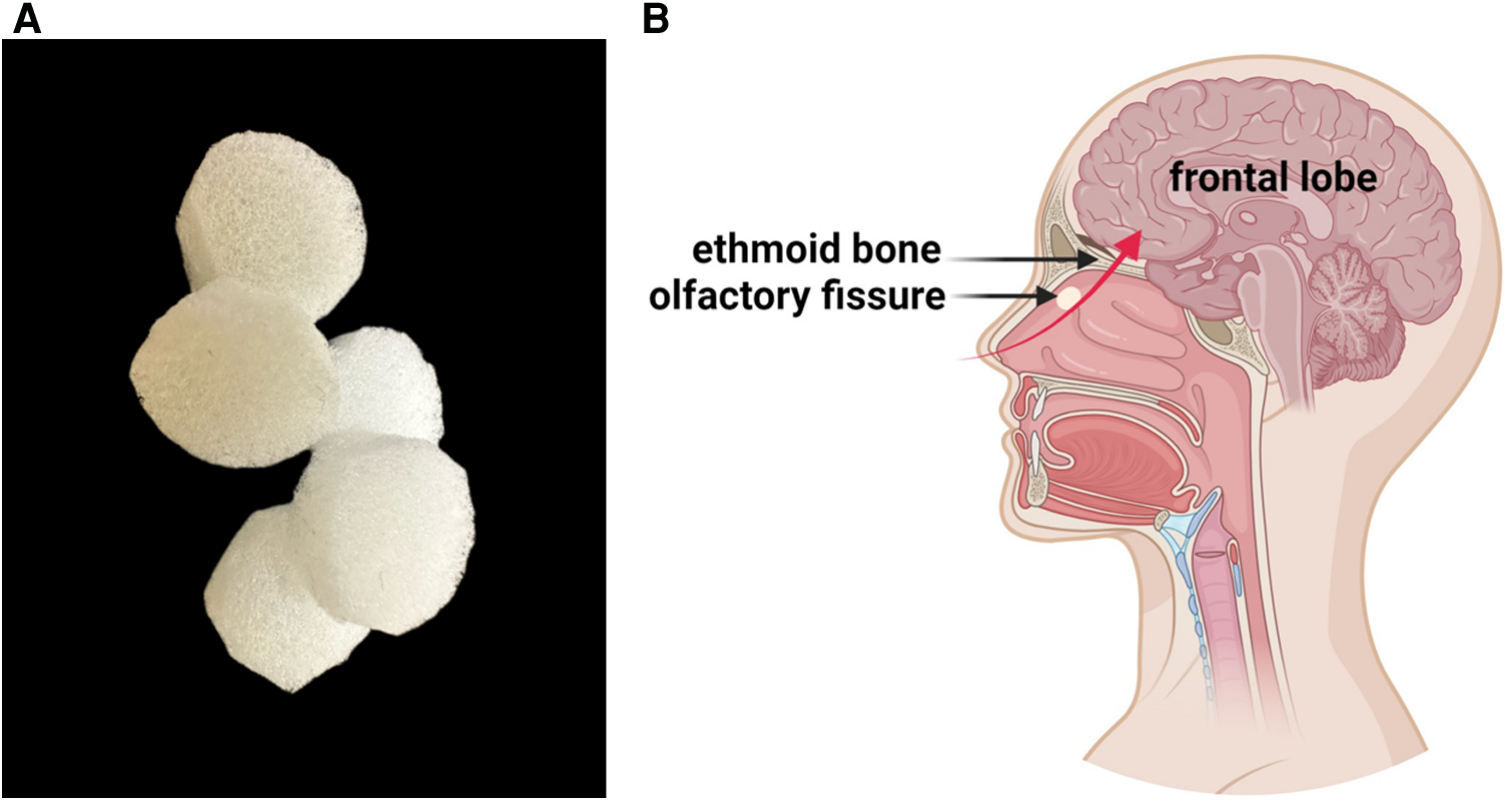
(**A**) Detailed view of the spongy material used to prepare the NSCs nasal patch. The material has a honeycomb pore structure that carries NSCs and can self-degrade in the body. (**B**) The patches are placed in the olfactory fissures of both nasal cavities. The red arrow represents the migration path of stem cells to the brain, with stem cells passing through the cribriform plate and entering the brain parenchyma.

A degradable NSCs preparation nasal patch was prepared using highly biocompatible honeycomb porous sponge material. It is the first non-invasive NSCs nasal patch used in international clinical research. It effectively breaks through the blood-brain barrier restrictions, and Non-invasive stem cells provide a new way to treat central nervous system diseases. A patent application has been submitted for this technology (International Patent No.: PCT/CN2019/077105; China Patent No.: PCT/CN2019/077105;201810623850.5) (14). The entire process of maintaining constant temperature and stable transportation to stem cell clinical institutions was performed in a special car. Otorhinolaryngologists used a disposable nasal drug delivery device to place a cut-sized patch containing NSCs (5 × 10^5 ^/kg) in the olfactory fissure of the bilateral nasal cavity of the child (Figure 1).

To reduce stimulation of the nasal cavity caused by patch transplantation, sedation and anti-allergic therapy were administered to the children step-by-step at 2 h, 1 h, and 30 min before transplantation. The clinical plan is as follows: Intravenous access was established 2 h before treatment, and loratadine was taken orally according to the child's body weight (children aged 2–12 years old: weight >30 kg, once a day, 10 mg; weight ≤30 kg, once a day, 5 mg). 1 h before treatment, 5 ml of cetirizine oral liquid was administered. Thirty minutes before treatment, 5 mg of dexamethasone was administered intravenously, and phenobarbital (0.2–0.4 mg/kg, extreme value ≤0.1 g) was injected intramuscularly according to body weight. 10% chloral hydrate enema (0.4–0.6 ml/kg, extreme value ≤1 g) was administered according to body weight. Before the enema, parents were advised to urge the child to defecate.

### 2.3 Statistics methods

Data were analyzed using SPSS 25.0. Count data are described by frequencies and percentages, and measurement data are described by means and standard deviations. The K–W rank sum test was used to compare the differences in the number of transplantations and nasal patch shedding in patients with CP. *P* < 0.05 was considered statistically significant.

## 3 Holistic care in the perioperative period

**Figure 2:**
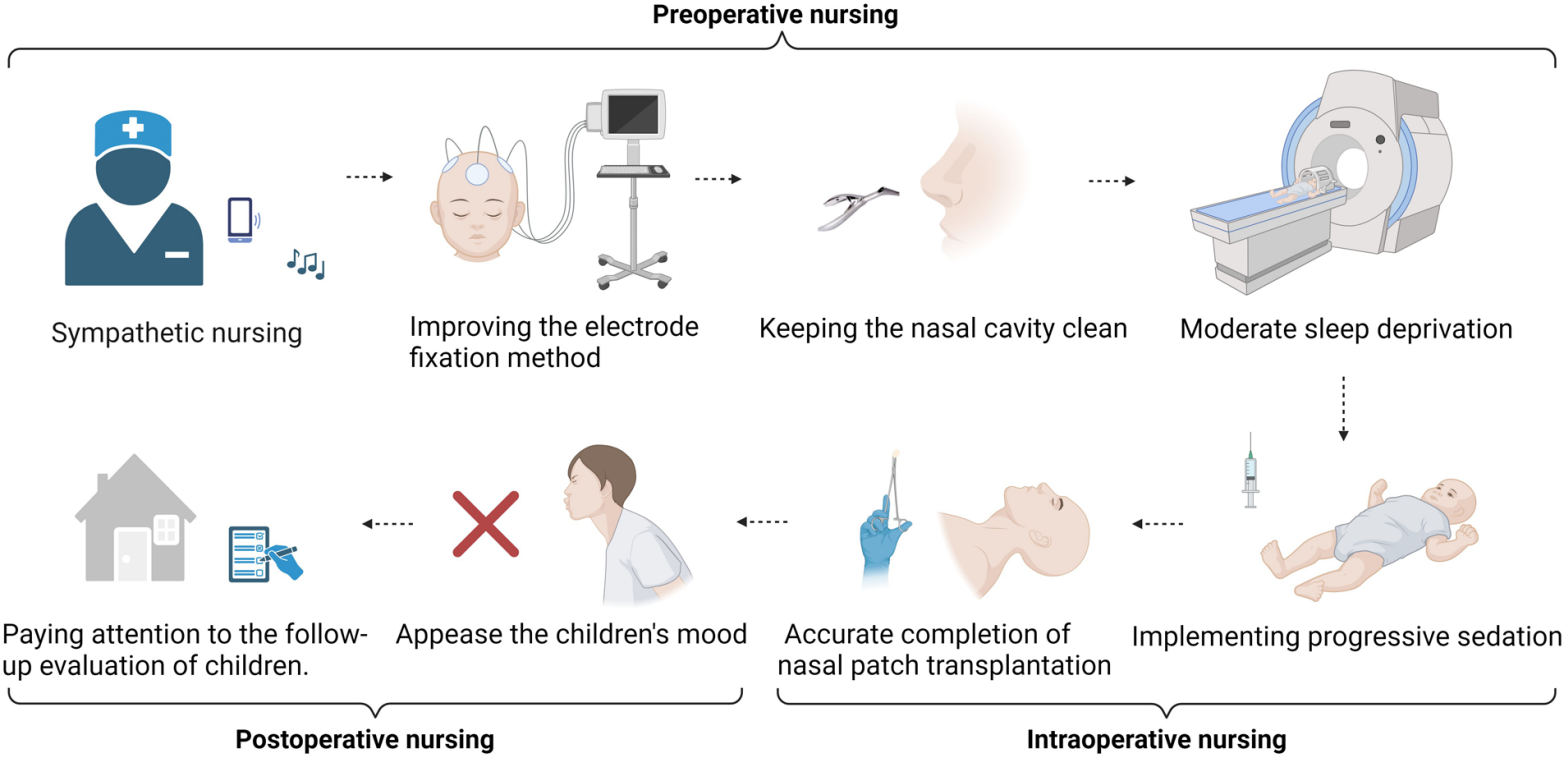
Preoperative nursing. Sympathetic nursing provides psychological support throughout the whole process. Improving the electrode fixation method to improve the accuracy and comfort of long-term ambulatory EEG examination. Keeping the nasal cavity clean and improving the success rate of stem cell transplantation. Moderate sleep deprivation to increase the combination of examination and treatment. Intraoperative nursing. Implementing progressive sedation to restrain the restlessness of children. Removal of pillows and horizontal posture fixation; accurate completion of nasal patch transplantation. Postoperative nursing. Integrating family affection and the emotional management of children after surgery. Ensuring the consistency of hospital, rehabilitation institution, and family care and paying attention to the follow-up evaluation of children.

By implementing holistic care with fixed persons, timing, and content, the perioperative nursing content was standardized, systematic, and professional, and the deficiency of routine nursing was avoided. The perioperative period of NSC transplantation for the treatment of CP requires the full participation of nurses. The unique status of nursing can enable the monitoring of the children's status in the perioperative period at any time and communicate key evaluation results to the team on time to provide management and support (Figure 2).

### 3.1 Preoperative nursing

#### 3.1.1 Sympathetic nursing provides psychological support throughout the whole process

CP has a low cure rate and a long disease course, and families require positive psychological support (21). The nursing team starts sympathetic nursing support throughout the process, understands the children's personalities and preferences from the caregivers in advance, tells stories, accompanies them to watch animated videos, and creates a positive, harmonious, and comfortable treatment environment. We introduce transplant procedures to children and their caregivers and fully inform them of potential risks and preventive measures. The WeChat official account, animated videos, and other ways to communicate about NSC transplantation technology allow children and caregivers to express their unknowns and discomforts and medical staff to give full understanding and respect as much as possible to allow them to receive NSC treatment in the best mental state possible.

#### 3.1.2 Improving the electrode fixation method to improve the accuracy and comfort of long-term ambulatory EEG examination

**Figure 3:**
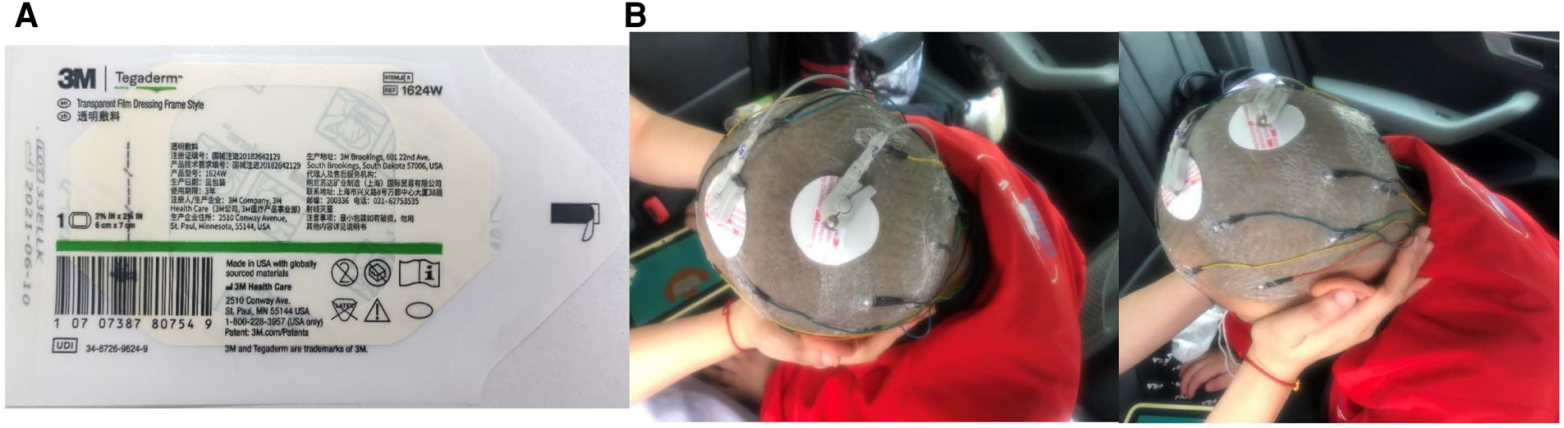
(**A**) Use tegaderm dressing to secure the EEG electrode pads to ensure connection stability. (**B**) Example of using Tegaderm dressing to immobilize a child with CP undergoing dynamic EEG examination.

This study used brain functional network energy analysis to evaluate changes in brain function before and after stem cell transplantation for the first time. 24 h AEEG examinations were performed before transplantation and at 24 h, 1 week, 1 month, 3 months, 6 months, and 12 months after transplantation. To ensure the accuracy of the evaluation data and the comfort of patients, the significance of dynamic EEG examinations, matters needing attention, and key points of nursing cooperation were introduced to patients and guardians to increase their enthusiasm and compliance. The caregiver was instructed to clean and shave the head within 24 h before treatment to ensure full contact between the electrode and the skin surface of the head. The electrode paste was applied to the electrode piece 1/3–1/2 to keep the scalp dry and paste successfully simultaneously; a Tegaderm dressing was used to fix it to ensure that the wire at the root of the electrode piece was pasted above 1.5 cm. To prevent a part of the electrode wire from falling off, it was strung into a bundle and fixed to the shoulder of the child. Finally, the head was wrapped with a double-layer elastic mesh cap to fix all electrode areas and ensure electrode stability (Figure 3).

#### 3.1.3 Keeping the nasal cavity clean and improving the success rate of stem cell transplantation

**Figure 4:**
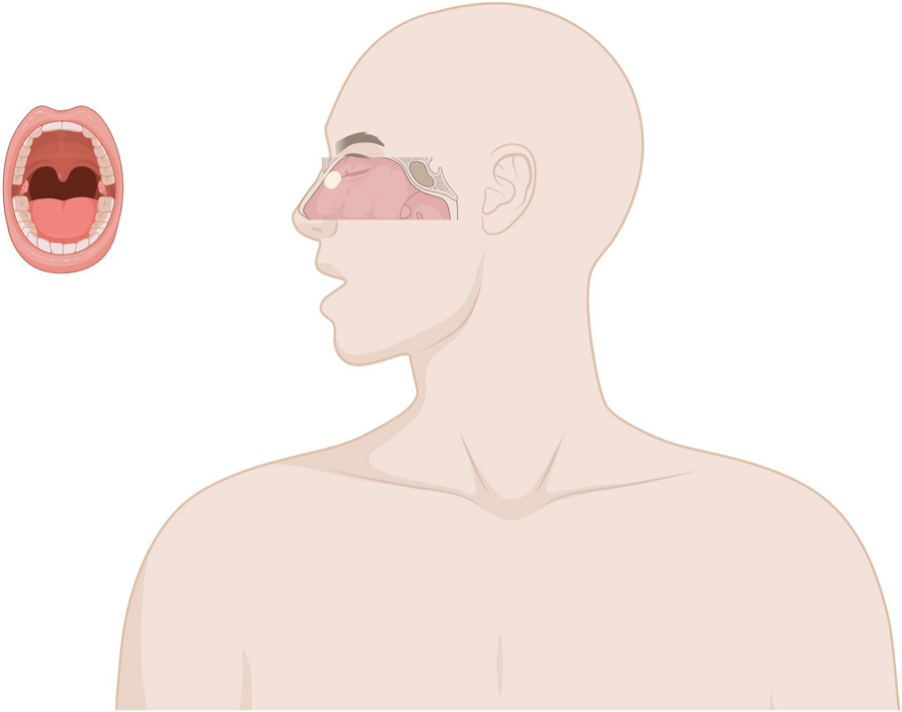
Diagram of mouth breathing. Instruct patients to practice mouth breathing throughout the transplant process.

Effectively reducing dirt in the nasal cavity reduces the number of colonizing bacteria in the nose and increases the success rate of stem cell transplantation (22). 3 days before treatment, a nurse assists the doctor in using the anterior nasal endoscope to examine the bilateral nasal cavity of the child and re-evaluates it 24 h before treatment. If there is a bleeding tendency, the treatment is suspended. On the day of treatment, a small, warm saline cotton swab is used to gently wipe the nasal cavity, soften and remove the sputum scab from the nose, improve the dryness of the mucosa, and keep the nasal cavity moist and comfortable. Relevant animation videos are provided to guide the children to practice open-mouth breathing and strengthen cooperation to avoid patch prolapse after successful transplantation (Figure 4).

#### 3.1.4 Moderate sleep deprivation to increase the combination of examination and treatment

Past trauma, hospitalization experience, and related cognitive levels undoubtedly determine the degree of examination and treatment for patients (23). Sleep deprivation is often used clinically to improve the degree of fit and sleep efficiency in children (24). Nurses can learn about the children's sleep habits from parents in advance, appropriately deprive them of sleep according to the situation, and encourage parents to reduce the length of sleep one day before treatment, such as by guiding children to go to bed 1 h later and get up at least 1 h earlier on the day of treatment. Simultaneously, earplugs of the right size should be selected on the day of examination and treatment to reduce external sound interference.

### 3.2 Intraoperative nursing

#### 3.2.1 Implementing progressive sedation to restrain the restlessness of children

Ideal sedation can reduce restlessness in children and enable effective, feasible, and safe transplantations (25). Nurses administer the medicine strictly following the clinical plan and complete the sedation and anti-allergic procedure for the children step-by-step at 2 h and 1 h before transplantation. While administering the enema, the child is placed in the left recumbent position, the hip is raised 20 cm, and the abdomen is cushioned with soft pillows. If the child has difficulty cooperating, one caregiver embraces the waist on one hand and the base of the thigh on the other, fully exposing the buttocks and fixing them properly. Nurses closely observe the status of children while simultaneously strengthening caregivers’ health education, improving their attention to light and digestible diets, and reducing the probability of adverse reactions.

#### 3.2.2 Removal of pillows and horizontal posture fixation; accurate completion of nasal patch transplantation

Proper head position and limited range of motion are necessary for intranasal administration (26). After successful sedation, the child is placed on a treatment bed to remove the pillow and lie flat. A nurse fixes the child's head slightly backward (the most important), with the doctor adjusting the angle of the head at any time; one assistant fixes the child's upper limbs and fore-chest, and another fixes the child's lower limbs. Attention is paid to gentle movements when fixed to reduce fear. Surgical anxiety is an important aspect of children's medical fear, and parental companionship can increase their compliance (27, 28). One parent was allowed to fix the child's upper limbs and fore-chest to comfort the child and reduce their alertness. Changes in vital signs, such as consciousness, blood pressure, pulse, respiration, and blood oxygen saturation, were closely observed during surgery. The surgery is stopped immediately and evaluated upon the occurrence of any adverse events, such as nasal bleeding.

### 3.3 Postoperative nursing

#### 3.3.1 Integrating family affection and the emotional management of children after surgery

The caregivers of the children are invited to participate immediately after the surgery, and the team members work together to appease the children's mood and display cartoons to divert their attention. Caregivers are instructed to help children breathe with their mouths open and reduce nasal breathing discomfort after patch implantation. The patch can be absorbed within 7–10 days after it is placed in the nasal cavity. If the patch falls off less than 4 h after transplantation, the doctor is immediately informed to retransplant accordingly. Medical staff and their caregivers monitor the status of the children, pay attention to the occurrence of fever, rash, nasal bleeding, and other adverse events, and deal with symptoms over time.

#### 3.3.2 Ensuring the consistency of hospital, rehabilitation institution, and family care and paying attention to the follow-up evaluation of children

The children who participated in the study required 3 months of stem cell transplantation treatment (once a month), 6 months of standardized rehabilitation, and no less than 12 months of follow-up evaluation. This process requires experimental centers, clinical departments, rehabilitation institutions, and families to complete the care of children. Nurses establish unified nursing concepts and methods with doctors and rehabilitators and provide standardized rehabilitation programs for children by distributing paper or electronic guidance manuals. An exclusive WeChat group of teams and family caregivers is set up to guide and answer their needs and questions in real time. Caregivers can directly send videos, such as walking, eating, reciting poems, holding objects with both hands, buttoning, and tying shoes, to the WeChat group or email.

## 4 Results

During hospitalization and follow-up, none of the children experienced adverse reactions related to immune rejection. However, one child developed dizziness, nausea, and vomiting after sedation. A small amount of bleeding of the nasal mucosa occurred in three children, and the temperature of 2 patients increased within 24 h and 2 days after transplantation (≤38.5°C). None of the patients had chills or a systemic infection. One child had a history of complex partial seizures (CPS), and the type and frequency changed after three months of NSC treatment. The patch fell off in three children within 4 h after being placed into the nasal cavity (Table 3). All patients underwent three transplantations to determine whether the bilateral nasal patches fell off within 24 h, and the results were *P* > 0.05 (Table 4).

**Table 3:** Adverse events.

Adverse events	Cases	Countermeasures	Results
Nausea, vomiting	1	Shortly after sedation, dizziness, nausea, and vomiting occurred during the examination. The nurses immediately helped remove the electrode of the lower right side of the head, removed vomit in the airway, appeased the child's mood, and avoided aspiration.	After vomiting, the symptoms of dizziness and nausea were relieved, and there was no fever.
Seizure	1	Three months after NSC treatment, the type and frequency of CPS seizures changed, and the dose of antiepileptic drugs (sodium valproate and carbamazepine) was adjusted.	The frequency of CPS decreased, and the disturbance of consciousness disappeared during CPS.
Nasal mucosa hemorrhage	3	Symptomatic treatment, such as compression, was given to stop the bleeding and protect the mucous membrane.	After compression, the bleeding stopped after 5 min.
Fever	2	According to the doctor's advice, acetaminophen 0.5 g should be administered, the body should be washed with warm water, and an adequate quantity of warm water should be taken.	After getting up the next morning, the body temperature returned to normal.
Patch removal within 4 h after transplantation	3	The internal diameter of the nasal cavity was measured, and the specification of individual patches was provided for children according to the actual measurement results.	The patch was re-implanted, and it did not come off.

NSCs, neural stem cells; CPS, complex partial seizures.

**Table 4:** Patch shedding within 24 h after transplantation.

Patch shedding location	First	Second	Third	*P* value
Both sides not	15	15	17	0.717
Only one side	4	1	4	
Both sides	3	6	1	

## 5 Discussion

This nursing experience comes from the team’s first international clinical research project on transnasal NSC transplantation for the treatment of CP (14). The research results have shown that transnasal NSC transplantation is safe and effective in the treatment of CP. This study, mainly based on the technical characteristics of transnasal stem cell transplantation and the needs of children with CP, formulated the full-process overall care and operating precautions before, during, and after surgery. While the treatment is safe and effective, it also increases the comfort of children and parents and proposes a comprehensive and novel overall care plan for children with neurological diseases after NSC transplantation.

Adverse stem cell-related events are closely associated with the route of drug administration. Although intrathecal, intravenous, or arterial injection is a common transmission route for stem cell therapy, preclinical studies have shown that nasal administration of stem cells, a promising cell delivery method for nervous system diseases, can effectively reduce the incidence of adverse reactions (29). It has been reported that fever is an expected adverse effect of stem cell transplantation (30). Therefore, we recommend that, after the completion of stem cell transplantation, the child should lie down and rest. Changes in body temperature should be closely monitored for 3–4 h, and detailed records should be made. If there are symptoms of respiratory tract infection, such as redness and swelling of the pharynx and a clear nose, the appropriate date of transplantation should be determined after a full evaluation to effectively avoid postoperative adverse reactions unrelated to stem cell therapy.

Treatment procedures, such as the placement of the NSC patch through the nasal cavity, cause anxiety and pain and lead to violent resistance and difficulty cooperating with the treatment. Some studies have shown that moderate sedation and sleep deprivation can effectively ensure the accuracy of examination results without motion imaging and smooth treatment progress (31, 32). The nurses in this study used phenobarbital, chloral hydrate, and other drugs to sedate the children gradually, according to the doctor's advice, and closely observed the status of the children during the sedation period. A few children will experience gastrointestinal irritation symptoms, such as nausea and vomiting, after taking chloral hydrate (33). Shortly after sedation, one child in this group showed symptoms of dizziness, nausea, and vomiting during the EMG examination, which were relieved after three episodes of vomiting. We suspected that the child's exposure to poor food hygiene and drug stimulation the day before the examination might have contributed to the symptoms. Due to the particularity of the disease, parents are the main caregivers of the children and simultaneously have feelings of pity and care for the children to meet all their needs (34).

To strengthen the caregivers’ cooperation and understanding of the transplantation work, the nurses issued a list of treatment instructions to them, introducing in detail the contents of each item in the manual. In addition to emphasizing the importance of a light diet before surgery, caregivers were also encouraged to be aware of the preparations and matters needing attention in every aspect of the children's hospitalization. Before sedation began, the nurses confirmed the diet and general condition of the children the day before surgery, which greatly reduced the occurrence of adverse reactions after sedation.

The 24 h AEEG used in this study compensates for the short recording time of routine EEG and can more comprehensively evaluate the changes in brain function of children before and after stem cell transplantation. The EEG electrode was fixed with a Tegaderm dressing, which can dissolve fibrin, maintain normal local tissue metabolism, reduce skin damage, have strong adhesion, and not be easily displaced (35, 36). The EEG examination methods adopted in this study successfully achieved image acquisition in children. Compared to the conventional method of fixing electrodes with collodion and other chemical reagents, stimulation to the children's scalp is reduced, the comfort and compliance of examinations are increased, and the installation and disassembly process is more convenient (37). Moreover, in the intensive care of premature infants, dressings that are ultrathin, soft, and can closely fit the baby's skin have been used in the skin electronic technology of wireless manipulation and power supply. This provides an important concept for developing flexible electrode technology for long-term EEG (38, 39).

Common adverse reactions after nasal administration include nasal burning, irritation, and other discomfort (26). Some studies have shown that isotonic solutions can increase the activity of mucosal cilia and effectively achieve nasal hygiene (40). The 2020 “Clinical Practice Guideline: Nosebleed (Epistaxis)” recommends that nosebleeds be filled with gauze or soft fabric to stop bleeding; sprays, ointments, and gels can be used to keep the nasal cavity moist; and persistent bleeding can be treated by arterial ligation or endovascular embolization (41). We thoroughly evaluated the nasal mucosa, provided appropriate moisturizing and cleaning treatment according to the condition of nasal dryness 3 days before the surgery, and repeated the evaluation 1 day before the surgery. Human epidermal growth factor was applied to the affected side of the nose three times daily to improve mucosal erosion. Additional bleeding can be treated with ice-salt water and norepinephrine-soaked gauze to stop bleeding while preparing pressurized hemostatic instruments to prevent nasal bleeding. No misentry of the patch into the airway was observed in this study. However, the patch was mistakenly inserted in the trachea in one case; the child was immediately placed in the lying position on the right side to remove the foreign body blocking the airway, and if necessary, a tracheotomy was performed to remove the foreign body. We also focused on shedding the patches. At the same time, the peeling off of the patch also attracts our attention. Table 4 shows *P *> 0.05, indicating that there is no significant difference between the patient's number of transplants and the nasal patch shedding. The degree of nasal patch shedding is not affected by the patient's number of transplants. It may be that the sample size is small and there is no significant difference. In the future, researchers can continue to analyze whether the difference between the two is statistically significant based on increasing the sample size. We should also pay more attention to the time and reasons for patch detachment and take more effective intervention measures to prevent patch detachment.

Modern medical management focuses on multidisciplinary cooperation, and the establishment of a multidisciplinary team combines expertise from different medical fields to provide comprehensive treatment advice for patients and facilitate decision sharing (42). This multidisciplinary team, dominated by nurses, realizes the four-in-one doctor-nurse-rehabilitation teacher-caregivers collaboration, which participates in the entire clinical research process, improves the safety of treatment and nursing, and further strengthens the follow-up management of children after discharge. The caregivers know the contact information and mailing address of the researcher, and we encouraged the caregivers to communicate directly with any team member or share the child's difficulties in treatment or rehabilitation on WeChat to ensure the child’s safety and reduce anxiety.

During follow-up, the type and frequency of CPS in one child changed, and the seizure frequency decreased after adjusting the dose of antiepileptic drugs. However, it is still unclear whether stem cell transplantation has a therapeutic effect on epilepsy or aggravates the disease (43, 44). In the future, healthcare workers still need to carry out more clinical research on stem cells, summarize the evidence for the safety of stem cell treatment for patients with epilepsy, understand the positive manifestations of epilepsy, effectively reduce the incidence of adverse events in children, and reduce the cost of medical treatment.

This was a single-center study; the current number of cases was limited; perioperative nursing programs were limited; and more studies from multiple centers need to be conducted in the future to verify and promote this program for the formulation of nursing guidelines in this field.

This study provides new and comprehensive nursing ideas for patients with CP who undergo transnasal transplantation of NSCs. Providing safe and effective sedation and nursing for children is an important prerequisite for the smooth progress of clinical work. Managing nasal conditions and the patient's emotions is also key to a successful transplant. Therefore, it is necessary to summarize the nursing process and precautions based on the characteristics of transnasal stem cell transplantation and the need for stem cell treatment for children with CP to provide a nursing reference for continued transnasal transplantation of NSCs to treat children with neurological diseases in the future.

## Data Availability

The original contributions presented in the study are included in the article/Supplementary Material, further inquiries can be directed to the corresponding author.
